# Near-field electromagnetic holography for high-resolution analysis of network interactions in neuronal tissue

**DOI:** 10.1016/j.jneumeth.2015.05.016

**Published:** 2015-09-30

**Authors:** Henrik D. Kjeldsen, Marcus Kaiser, Miles A. Whittington

**Affiliations:** a University of Newcastle, Institute of Neuroscience, Framlington Place, Newcastle upon Tyne NE2 4HH, UK; b University of Newcastle, School of Computing Science, Interdisciplinary Computing and Complex BioSystems Research Group, Claremont Tower, Newcastle upon Tyne NE1 7RU, UK; c University of York, Hull York Medical School, York YO10 5DD, UK

**Keywords:** Electromagnetic field, Holography, Microelectrode array, Dynamic causal interactions

## Abstract

**Background:**

Brain function is dependent upon the concerted, dynamical interactions between a great many neurons distributed over many cortical subregions. Current methods of quantifying such interactions are limited by consideration only of single direct or indirect measures of a subsample of all neuronal population activity.

**New method:**

Here we present a new derivation of the electromagnetic analogy to near-field acoustic holography allowing high-resolution, vectored estimates of interactions between sources of electromagnetic activity that significantly improves this situation. In vitro voltage potential recordings were used to estimate pseudo-electromagnetic energy flow vector fields, current and energy source densities and energy dissipation in reconstruction planes at depth into the neural tissue parallel to the recording plane of the microelectrode array.

**Results:**

The properties of the reconstructed near-field estimate allowed both the utilization of super-resolution techniques to increase the imaging resolution beyond that of the microelectrode array, and facilitated a novel approach to estimating causal relationships between activity in neocortical subregions.

**Comparison with existing methods:**

The holographic nature of the reconstruction method allowed significantly better estimation of the fine spatiotemporal detail of neuronal population activity, compared with interpolation alone, beyond the spatial resolution of the electrode arrays used. Pseudo-energy flow vector mapping was possible with high temporal precision, allowing a near-realtime estimate of causal interaction dynamics.

**Conclusions:**

Basic near-field electromagnetic holography provides a powerful means to increase spatial resolution from electrode array data with careful choice of spatial filters and distance to reconstruction plane. More detailed approaches may provide the ability to volumetrically reconstruct activity patterns on neuronal tissue, but the ability to extract vectored data with the method presented already permits the study of dynamic causal interactions without bias from any prior assumptions on anatomical connectivity.

## Highlights

• We developed a method to estimate electromagnetic field vectors from microelectrode array data.
• The vectors allow high-resolution holographic reconstruction of spatiotemporal activity.
• Separation of electromagnetic source density and dissipation informs on activity structure.
• Electromagnetic flow maps quantify dynamic causal interactions in brain tissue.

## 1 Introduction

Neuronal function, from an electrical point of view, originates from the control and utilisation of current flow across biological membranes. A myriad of different proteins are incorporated into membranes to create a baseline ‘set point’ for neuronal membrane current flow and provide an incredibly rich diversity of magnitudes and kinetics of deviations from this. These currents can be quantified directly in individual neurons by invasive techniques utilising direct electrical access to the intracellular environment of individual – or small subsets of – neurons. However, neurons do not act alone: They are embedded in specific local and distributed networks allowing them to influence each other's activity and act in concert to represent external (sensory) events and generate appropriate, patterned outputs (motor).

It is increasingly evident that cortical activity at the level of interacting populations of neurons holds the key to understanding brain function (Wetzel and Stuart, 1976). Therefore, in considering population-level neural behaviour one must consider interaction across the whole neuropil and whatever reciprocal, synaptic interactions with neurons emerge from this (Mitzdorf and Singer, 1979). However, there are two additional fates for the energy associated with changes in neuronal transmembrane current flow: First, changes in charge distribution across membranes lead to electrical potential energy changes organised spatially within the extracellular environment (the local field potential, LFP). These fields can feed-back to influence neuronal activity directly (Taylor and Dudek, 1984); Secondl, the same transmembrane charge distribution changes can give rise to magnetic fields. While these are much weaker than the electric fields they may also feedback to influence neuronal transmembrane current, at least over very short distances (McLean et al., 1995), unless fields are artificially large (Houpt et al., 2003).

In attempting to further understand the entirety of the electromagnetic interactions between brain regions, and link this to the causal dynamics of the system we noticed that similar problems have been addressed in acoustic imaging, specifically in near-field acoustic holography (NAH) (Maynard et al., 1985; Thomas et al., 2010). We therefore set out to explore whether analogies between acoustics and electromagnetics could be used to generalize this technique to the neuro-electromagnetic case, i.e. to near-field electromagnetic holography (NEH). The idea of NEH goes further than considering the activity recorded in electrodes used to study neuronal populations: By considering recorded activity as a map of the electromagnetic interference between signals originating from a set of sources an estimate of these original sources can be reconstructed via holographic methods. This method has potential advantages over existing source localisation methods for neural electrical activity. Whilst this approach has been shown to be valid for a few coexistent sources (Alqadah et al., 2014) it needs to be applied to sensory (microelectrode) arrays to be useful in localising and characterising the many multiples of activity sources that typify population neuronal activity. For example, conventional current source density (CSD) estimates map the origin of activity by considering only the *average* of sources in a given locale. In contrast, a holographic reconstruction, by being dependent on interference between sources may not suffer as much from this inherent averaging effect—thus a greater spatial resolution of multiple source structures should be possible (see Maynard et al., 1985).

In addition, source reconstruction using acoustic holography works through the reconstruction of acoustic energy flow (Hald, 2001). The electromagnetic analogy of this is the Poynting vector, where the scalar is the electromagnetic energy flux density and the direction represents the flow from source to sink. That is for a given source region the vector points in the direction of the mean, largest recipient of the electromagnetic energy estimated (Williams and Maynard, 1980). Considering that causal effects of one population of neurons on another must reasonably be carried by physical energy flow, whatever the conduit, we propose that electromagnetic energy flow vectors can be used to infer causal effects in neural tissue with fewer of the biological and statistical assumptions required for methods used presently.

However, for accurate reconstruction of sources nearfield holography requires back-propagation of recorded signals through a homogeneous, source-free medium (i.e. no additional sound or electromagnetic generators between the electrode array and the reconstruction plane of interest (Valdivia and Williams, 2007)). While compensation for lack of homogeneity can improve CSD estimates of electrical sources (Lęski et al., 2011), it forms a major problem with acoustic sources (Williams and Valdivia, 2010; Bi et al., 2015). This suggests a uniquely appropriate application for NEH on cortical tissue: Brain electromagnetic sources have no ‘physical’ structure in the sense that they do not overtly modify the signal conduction properties of the medium unless they become excessive (for example in epilepsy). Resistivity in neuronal tissue *does* change with activity, but with a relatively slow timeconstant (5–10 s, Fox et al., 2004; Lopez-Aguado et al., 2001)—far slower than even the slowest brain activity studied with conventional EEG and local field potential recordings. In addition, permittivity has been shown not to change at all during intense periods of electrical activity (Yoon et al., 1999).

The problem of holographic reconstruction in media with multiple sources requires inhomogeneous wave equations to be used to reach a precise, accurate answer. While this approach works numerically with known additional sources this luxury is not afforded by studies of brain tissue: here each microscopic component of the neuropil may constitute a source at any given time. The down-side of this is that any reconstruction will only ever reflect an *estimate* of the true nature of sources present. On the other hand, the ubiquitous, distributed nature of additional sources provides a blanket ‘forcing’ of the wave equations required. As a consequence the holographic reconstruction becomes probabilistic, with only the largest, most spatially focal sources of activity surviving the process. An additional implementation problem arises owing to the fact that the electrode arrays used for invasive recording are effectively embedded within such a heterogeneous, spatially-distributed source. This invalidates the use of holographic reconstruction in terms of providing *absolute* physical quantities such as electric and magnetic fields. However, the use of estimates of orthogonal partners to the measured electrical field (the estimated magnetic field) still provides a valid means to vectorise the activity present and therefore attempt to improve spatial resolution of source distribution through NEH. Thus the myriad potential sources present in active neuronal tissue, and the relatively static, homogeneous nature of the transmission properties of neuropil (even with active sources) on physiologically relevant timescales suggests that NEH may provide an improved means to estimate spatiotemporal activity patterns in the brain.

Here we test this hypothesis and present a method for analysing extracellular microelectrode array recordings. We utilise the Poynting vector to estimate the electromagnetic energy transfer per unit area of neuronal tissue. In doing so we are able to consider a direct analogy of current source density—the *electromagnetic energy source density* alongside *electromagnetic energy dissipation* (the proportion of charge distribution that is dissipated rather than contributing to the magnetic field) to provide a ‘super-resolution’ estimate of energy flow between brain sub-regions as a vector. We test this method by demonstrating the improved spatial detail and highly directional behaviour of a known, highly laminarly organised spatiotemporal dynamic phenomenon—the sleep-associated slow wave oscillation in an in vitro experimental model.

## 2 Methods

NAH typically involves the use of planar arrays of microphones (Paillasseur et al., 2011). For an implementation with electromagnetic sources from brain tissue the following precedented analogies (Morgan, 2003) from other NEH implementations are helpful (see Table 1): Microphone arrays corresponded to microelectrode arrays. Acoustic pressure and particle velocity corresponded to the electric-field and the magnetic-field, respectively, and acoustic intensity corresponded to the Poynting vector (Williams and Valdivia, 2010). There is also an important analogy between the acoustic properties of the acoustic medium, and the complex conductivity, permeability and permittivity of the neural tissue (Nicolas et al., 1998). We obtained the relevant conductivity values from direct measurements in rodent neocortex given in the literature by layer, and across and along layers. In conductive conditions the acoustic equations carry over directly, only now with complex wave numbers, and electromagnetic instead of acoustic interpretations (Goto et al., 2010). The near-field is characterized by evanescent, exponentially decaying waves of high frequency, which contain detailed spatial information that is unrecoverable in the far-field giving rise to the well-known diffraction limit on imaging resolution. However, when measured sufficiently close to the sources (‘near-field’) and sampled at a sufficiently high rate the evanescent waves can be recorded and included in the holographic reconstruction allowing recovery of detailed spatial information for resolution beyond the resolution of the sensor array (Williams and Maynard, 1980).

**Fig. 1:**
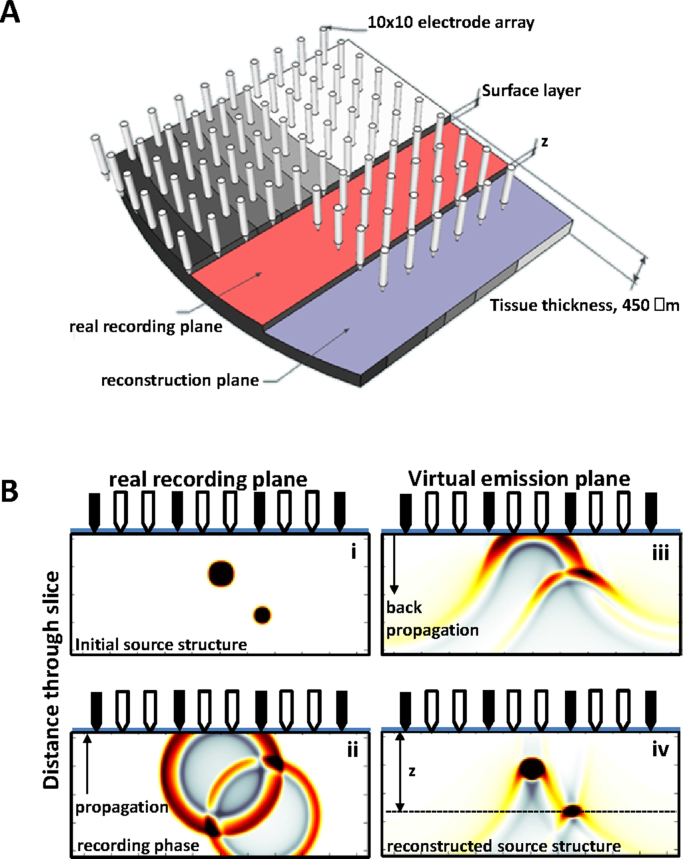
*Basic application of near-field electromagnetic (NEH) holography*. (A) Cartoon illustrating the data collection paradigm and its relation to the near-field dimensions used. Recordings of neocortical tissue voltage were taken with a 10 × 10, square array of silicon electrodes with inter-electrode distance of 0.4 mm. Arrays were placed on the upper cut surface of the tissue (the real recording plane in the figure) with the leftmost column of electrodes aligned to the pial surface. The reconstruction plane used to estimate electromagnetic properties of on-going activity in the tissue was set between ca. 65 and 265 μm into the slice (distance *z*) to obtain optimal spatial resolution (see Fig. 3). (B) Example of electromagnetic field source reconstruction using the experimental approach outlined in (A) simulated using the K-Wave toolbox (Treeby and Cox, 2010). Two circular sources of differing size and distance from the slice surface (real recording plane) were simulated. (ii) Electrical activity projecting from these sources was recorded as voltage change by the nearby electrodes (black arrows) and interpolated to two virtual electrode points (white arrows) between each real electrode using the Papoulis–Gerchberg algorithm [12]. (iii) Real and interpolated data is projected back into the tissue from the recording plane (now a virtual emission plane) using a k-space propagator and filter (see text) to estimate the direction and intensity of electromagnetic energy flow (Poynting vector, Fig. 2) from tissue properties. (iv) Accurate source structure reconstructed using this holographic process is dependent on the physical relationship between original source location and the chosen reconstruction plane.

To explore the practical usefulness of this method with electromagnetic data we used planar Utah arrays consisting of 10 × 10 electrodes with orthogonal separation of 0.4 mm placed on the surface of an isolated slice (450 μm thick) of neocortical tissue (Fig. 1A). Recordings of voltage changes (0–30 kHz) in the neuropil during an in vitro model of neocortical delta rhythms (1–4 Hz Carracedo et al., 2013) were used as the starting point of the holographic reconstruction. These rhythms, like their in vivo counterparts during deep sleep, are relatively stationary for many tens of minutes to several hours, thus permitting the use of the Fourier transform-based approach to source reconstruction (e.g. see Thomas et al., 2010). Fig. 1B shows the basic principles of the reconstruction process. Initially, electromagnetic field sources in the active neural tissue project outward and generate voltage changes detected by the electrode array in the real recording plane. This ‘real’ data was first interpolated to prepare for super-resolution (see below) using the Papoulis–Gerchberg algorithm (Papoulis, 1978), a procedure shown to enhance resolution of NAH reconstructions previously (Xu et al., 2008). We increased resolution via interpolation 3 times in each direction though the limits of the effect were not explored.

**Fig. 2:**
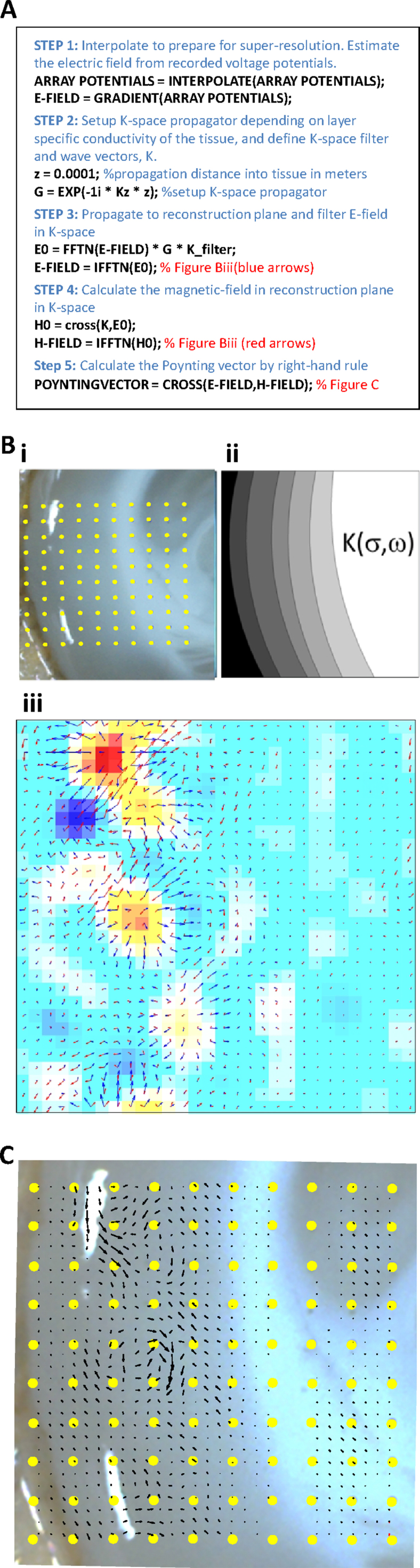
*Calculating the Poynting vector from the electric (measured) and magnetic (estimated) fields*. (A) Extracts of pseudocode highlighting the steps required to generate the electromagnetic energy flow in the reconstruction plane. Electric and magnetic fields were estimated at 0.1 mm (*z*) from the real recording plane (tissue surface) by applying a k-space propagator and filter constructed using published values of conductivity, permeability and complex permittivity through neocortex. (B). (i) The location of the real recording electrodes in the arrays used relative to a slice of neocortical tissue maintained in an interface chamber (see text). (ii) The corresponding basic structure of the layer-specific electromagnetic properties used to build the k-space filter for back-propagation. (iii) The resulting estimates of electric field (blue arrows) and magnetic field (red arrows) in the reconstruction plane (set in this example 0.1 mm from the real recording plane) overlaid on the interpolated voltages (red positive, blue negative relative to rest) taken from the recording plane. Arrow length represents local field strength, arrow direction represents local field propagation vector. (C) Overlay of the reconstructed electromagnetic energy flow in the reconstruction plane onto the array of real electrodes and the tissue recorded from. Arrows represent the local energy flow direction and intensity (angle and length of arrows, respectively). Note the strength of the electromagnetic energy flow does not map linearly onto the original recorded voltages and the units for each of the reconstructed properties plotted are arbitrary (i.e. they do not directly map onto the ‘real’ EM field properties at source). (For interpretation of the references to color in this figure legend, the reader is referred to the web version of this article.)

The reconstruction process required to obtain Poynting vector estimates is summarised as pseudocode in Fig. 2A (and in detail in the table of formulae used (Table 2)) and were as follows: First, the electric field was calculated from the recorded voltage potentials. This was then propagated in k-space (the spatial-frequency domain, see Morgan et al., 2003 for single sensor implementation) to the reconstruction plane to give an electric field estimate based on wave-vectors constructed using the published electromagnetic properties of neuronal tissue (Fig. 2Bii). In this way both the original electrode voltage data (positions shown in Fig. 2Bi) and the interpolated data are acted upon in the same manner to give a higher-resolution picture of the electric field within the slice at the chosen reconstruction plane (Fig. 2Biii). From this the ‘magnetic field’ was estimated, again taking into account the electromagnetic properties of the tissue inherent in the k-space filter and assuming no derangement of distal source activity by more proximal sources (see Fig. 1B and Section 1). As the recording aperture in this example is smaller than the source medium we used zero-padding as a simple way to alleviate spectral leakage. More rigorous methods have been reported (e.g. Liu et al., 2011) but were omitted for reasons of computational tractability. The Poynting vector field was given by a right-hand rule from these reconstructed electric and magnetic fields (Fig. 2C). All analysis was performed using Matlab (The Mathworks, Natick, USA) and code is available from the authors on request.

**Fig. 3:**
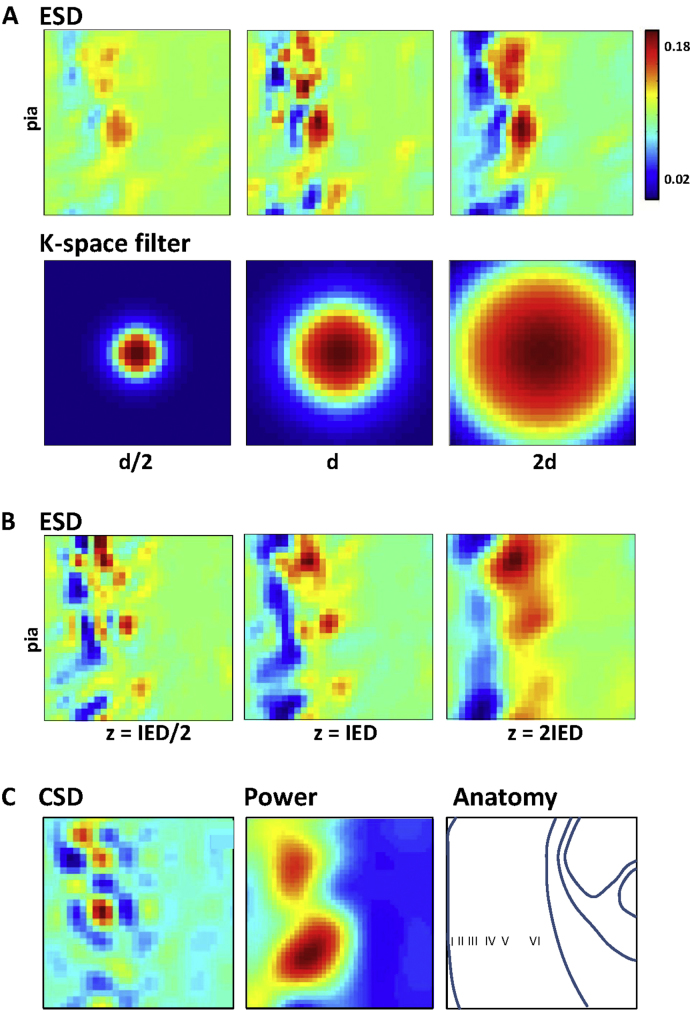
*Effects of k-space filter width and recording/reconstruction plane separation on estimated energy source density structure*. (A) Energy source density (ESD) maps for different k-space filter widths (d). Note ESD is the electromagnetic correlate of current source density and is generated from the divergence of the Poynting vector. The degree of spatial detail increases with finer spatial filtering until reconstructed activity begins to disappear into noise. (B) The spatial scale at which this occurs is dependent on the relative values of interelectrode distance (IED) and distance from recording to reconstruction plane (*z*). (C) Comparison with conventional activity maps. Current source density (CSD) reveals discrete source/sink loci but fails to capture the laminar organisation of the delta rhythm-related activity. Power maps (raw delta power from Fourier transform of the recorded voltage signal per electrode) reveal the deep layer domination of this activity but not the overt laminar differences revealed by ESD. Right panel shows a crude representation of the cortical anatomy within which the activity is recorded/reconstructed.

Clearly the performance of the NEH reconstruction process depended a great deal on the spatial constraints the k-space filter contains. In assessing this it was useful to consider the divergence of the Poynting vector field. Visualising source and sink locations within the reconstructed electromagnetic field (the Energy Source Density, ESD) gave not only a clear demonstration of the laminar nature of the slow wave behaviour being studied, but also a means to optimise the filter characteristics (Fig. 3). Altering the spatial frequency of the filter could ‘focus’ the reconstruction to optimise the spatial resolution of the NEH process. However, too-tight a filter resulted in loss of signal within the overall noise of the system (Fig. 3A, leftmost panel). Similarly, the relationship between array electrode separation and the distance from real recording plane to reconstruction plane (*z*, see Fig. 1) was critical for optimal reconstruction. Greater distances generated more ‘blurred’ spatial patterns of ESD, whereas reconstructing too close to the recording array generated highly fractured ESD spatial profiles (Fig. 3B). Despite this, considering the reconstructed ESD revealed a clearer laminar structure to the activity in neocortex than obtained by conventional current source density or raw power maps alone (Fig. 3C).

## 3 Results

**Fig. 4:**
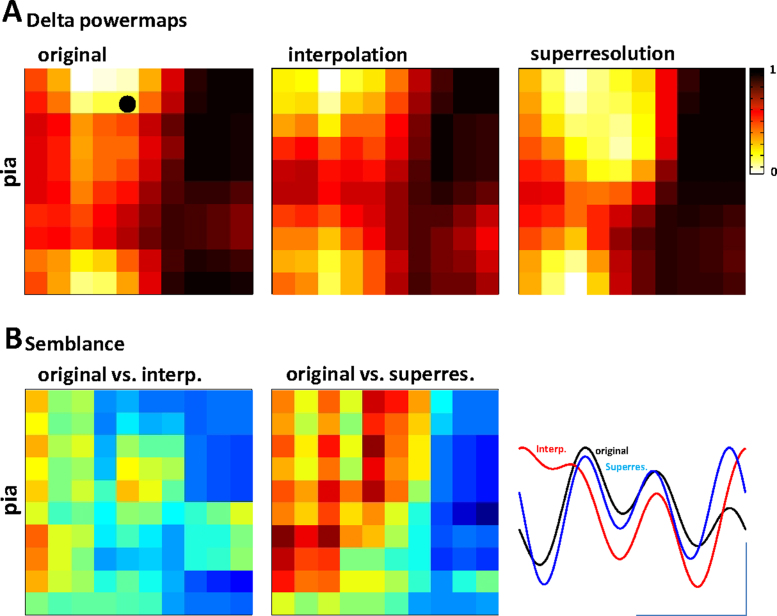
*NEH superresolution is significantly superior to interpolation alone in reconstructing spatiotemporal properties of the delta rhythm*. (A) Example powermaps of delta activity recorded with a 10 × 10 electrode array. Left panel shows delta power (0.5–2 Hz) derived from the FFT of the original voltage recording. Middle panel shows the delta power derived from the NEH method after halving spatial resolution by taking data only from every other electrode and inserting virtual electrodes as in Fig. 1A. Right panel shows delta power derived from interpolation of data from every other electrode in the original dataset. B. Wavelet semblance (Grinsted et al., 2004; Cooper and Cowan, 2008) maps of the two methods and comparison of 3 periods of delta rhythm for the same electrode/reconstruction vs. the original data (black circle in A). The spatiotemporal accuracy of the NEH superresolution was significantly better than interpolation (*P* = 0.0108).

Two main advantages are apparent in using NEH over existing methods to spatially map activity within brain regions and the causal interactions between them. First, in considering both electrical and magnetic behaviour the holographic method should, in principal, provide a better estimate of the spatial structure of activity beyond the resolution of the real recording electrode array. To test this, power maps of delta activity, derived from the FFT of the original data in a 10 × 10 array (Fig. 4A), were compared with those derived from 2-fold spatially downsampled original data (i.e. taking data from only every other electrode: a 5 × 5 array). Interpolation of the original downsampled data, back to a 10 × 10 resolution produced inferior results to interpolation modified through the NEH reconstruction. This was apparent both in the spatial maps of delta power (Fig. 4A) and in the similarity of the reconstructed timeseries data using phase semblance (Grinsted et al., 2004; Cooper and Cowan, 2008). Fig. 4B illustrates an example of the original, interpolated and NEH-reconstructed timeseries for a single electrode on the array, and semblance maps of these comparisons between the original data and the interpolated or NEH-reconstructed data. Taken for all matrix elements in the semblance reconstruction the performance of NEH reconstruction was highly significantly superior to interpolation alone (*P* < 0.02).

**Fig. 5:**
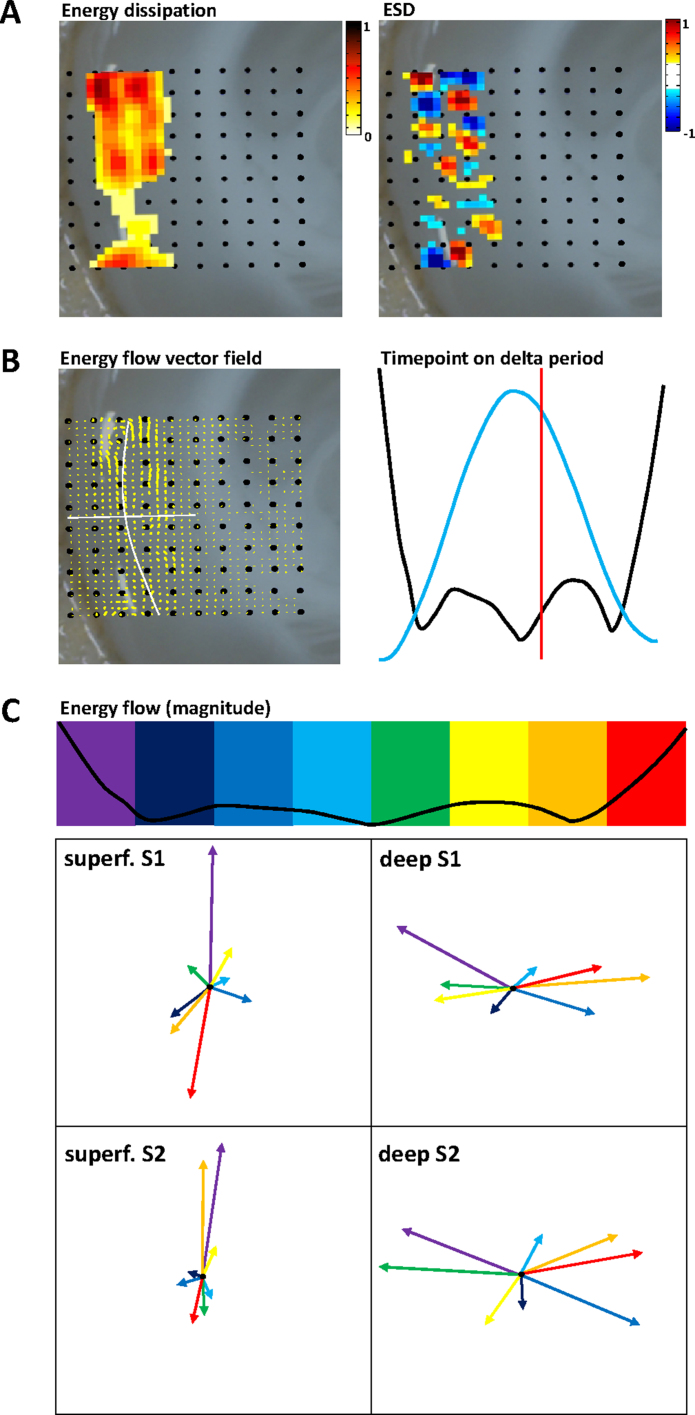
*NEH reveals lamina-specific structure of spatial interactions nested within each period of the delta rhythm.* (A) Estimation of the combined electromagnetic behaviour of neural activity from multiple periods (*n* = 60) allows the separation of the reconstructed signal into that which is ‘lost’ in terms of local activity in the system (energy dissipation, upper left panel) and that which is propagated within the tissue (energy source density, upper right panel). Note, energy dissipation reflects the predominantly infra- and supragranular laminar structure of the delta rhythm (e.g. Carracedo et al., 2013) whereas source density (ESD) reveals a ‘chequerboard’ spatial structure in which regions propagate activity to their near neighbours both horizontally and radially in neocortex. Note the colorbar scales for dissipation and ESD have arbitrary units. (B) These local interactions, derived from the vector field (upper left panel) can be studied at discrete timepoints by plotting the magnitude (black line, upper right panel) against the original, low pass filtered average delta period (blue line). Note the timepoint indicated by the red cursor corresponds to the vector field shown in the left panel. (C) Poynting vectors averaged in 4 regions (deep and superficial primary (S1) and secondary (S2) somatosensory cortices (see (B) left panel for regional separation)) at 8 timepoints within an average delta period. The combined electromagnetic nature of the tissue-generated activity revealed a dichotomy between supra and infragranular layer interactions: During delta rhythms infragranular layers projected their energy predominantly interlaminarly whereas supragranular layers projected horizontally. In both cases at least two cycles of vector change were seen nested within each average delta period.

Second, in reconstructing a vector field it was possible to quantify the directionality of the electromagnetic signals estimated through NEH reconstruction (Fig. 5). This novel approach allowed the visualisation of interactions between brain areas with: (a) no confounding statistical problems generated by many-multiple pairwise comparisons (Zou et al., 2010); (b) no reliance on accurate autoregressive modelling of the original data (Dhamala et al., 2008); (c) near-realtime quantification of any changes in directionality in the influence of one area over another. In addition, the measures of energy dissipation and energy source density in terms of source/sink location and magnitude could be considered separately from the electromagnetic causal directionality (Fig. 5A). In doing so, fine temporal and directional structure was observed within each delta period. Average energy dissipation per delta period revealed nested higher frequency changes in activity (Fig. 5B) which, in turn, corresponded to multiple, highly directional inter-areal interactions between superficial layers of primary and secondary somatosensory cortex in the tissue, coupled with highly interlaminar interactions dominating in deep layers within each cortical region (Fig. 5C).

## 4 Discussion

An estimate of energy flow within neural tissue in general has tremendous advantages over other estimates of causal interactions between brain areas as the latter rely on statistical analysis of variance between many-multiple pairs of microelectrodes. These are inherently far removed from the underlying neural activity, and also easily become intractable when the number of microelectrodes is high. In addition, in considering the tissue as a whole no prior assumptions have to be made regarding which regions constitute ‘nodes’ in whatever network may be active. Using the NEH process as described it was interesting to note that, even with no prior assumptions on connectivity structure, electromagnetic energy magnitude and flow estimates validated the interlaminar neocortical interactions and cross frequency interactions previously demonstrated by highly invasive (single neuron), detailed modelling and experiment (Carracedo et al., 2013; Hughes and Crunelli, 2013).

However, it is still early days for the NEH technique, and further study and reproduction is needed to verify its validity. It is clear that both conceptual and implementation issues are possible at this stage, and it is known from acoustics that many different implementations are possible with different advantages; for instance the known limitations of the Fourier transform can be mitigated by data padding (Hald, 2001), or be replaced by time domain (Zhang et al., 2011) or wavelet approaches (Thomas and Pascal, 2005). In the application presented here it was clear that care was needed to ‘focus’ the reconstruction plane on a potential cohort of sources. Failure to match the parameters for the k-space filter with the distance between the electrode (measurement) plane and the desired reconstruction plane resulted in blurring or breakdown of the source structure detected (Fig. 3) to a point where conventional methods gave better results. This is in part due to the non-passive nature of the projection medium: i.e. what separates the electrodes from the reconstruction plane is neural tissue which is also likely to contain further sources (e.g. Williams, 1999). More recent approaches have suggested a method to minimise this – and thus quantitatively optimise k-space parameters for a given distance between recording and reconstruction planes – by comparing the difference in relative magnitudes of the contribution of propagated and evanescent waves at the two planes (Tang et al., 2012).

The good performance of this initial implementation (compared to conventional CSD approaches) suggested that sources between the electrode and reconstruction planes did not overtly derange the holographic reconstruction process—a predicted outcome given the minimal, if any, effects of time-discrete electrical activity on the macroscopic resistivity and permittivity of neuropil (Lopez-Aguado et al., 2001; Fox et al., 2004; Yoon et al., 1999). The ‘forcing’ effect of additional sources on the numerical reconstruction process constrains the results of NEH reconstruction to only ever constitute an estimate of the true source structure in the tissue. However, when there is no a priori knowledge of the explicit source structure, and that source structure is highly distributed and complex (as in the case of active neuropil), this estimate become probabilistic: the reconstruction process will reveal only the largest, most spatially constrained activity patterns. As the very nature of cortical function is suggested by many to be based on probabilistic processes (e.g. see Rao et al., 2002) we see this caveat of the current method to be advantageous. In addition, the basic nature of the reconstruction algorithm used may also contribute to this process: As data in Fig. 3 show, NEH in this implementation is not a tomographic process (i.e. cannot be used for volumetric reconstruction), because the back-propagation technique is essentially a 2D-2D model, and a 2D-3D model is not unique (Devaney, 1978). This means that any reconstruction plane will exhibit some influence from sources in nearby planes, and therefore more resembles a local depth average than a sharp focal plane. The reconstruction plane (and the back-propagation filter) must therefore be picked heuristically to produce the best image given the specific circumstances.

It might be also be particularly important to consider that the energy flow estimates obtained with the present holographic approach are complex valued, and may therefore require a more physical interpretation. In NAH the real part of the energy flow estimate is associated with propagating energy waves whereas the imaginary part is associated with resonant standing waves (Mann et al., 1987; Domenico et al., 1996). In the electromagnetic case we can therefore propose an analogous interpretation with the imaginary part reflecting resonant energy transfer (a known near-field effect Lipworth et al., 2014). This view would suggest that we are not only dealing with a neural circuit in the conventional synaptic connectivity sense, but also a neural antenna network (as the closest electrical engineering analogy), where ‘passive’ propagation of electromagnetic energy through the neuropil also contributes to neuronal population activity patterns through ephaptic coupling (Jefferys, 1995).

In acoustics it is sometimes advantageous to record the particle velocity instead of the acoustic pressure, with the analysis proceeding in the same way; the electromagnetic analogy would then be to record the magnetic field instead of the electric potentials as is done in MEG (Cohen, 1968). It is also currently possible to record both electric and magnetic consequences of neuronal population activity concurrently (Tallon-Baudry et al., 1997), potentially reducing the number of estimation steps required to derive the Poynting vector. While basic calculations of optimal recording-reconstruction plane distance (Tang et al., 2012) indicate this is not possible with currently-employed superconducting detectors, newer ‘room-temperature’ detectors that are locatable on the scalp may generate data that is amenable to NEH. Further testing of this analytical approach is required. Finally, a more tomographic approach may be feasible for large electrode array recordings with implementation of ‘compression’-based reconstruction algorithms (Alqadah et al., 2014) However, the results presented here demonstrate that a relatively simple reconstruction method for NEH-derived vector fields can already provide a highly useful, novel means to study the dynamics of causal interareal interactions in the brain in the absence of prior assumptions about network structure.

## Figures and Tables

**Table 1:** Analogous properties and nature of the components required for holographic reconstruction.

NAH	NEH	M/E	NEH calculation
Acoustic pressure	Electric field	M	Spatial gradient of recorded voltage potentials
Propagation from microphone/electrode array to the reconstruction plane		M/E	*Inverse Fourier transform of spatial fourier transform of electric field and propagator (k-space filter)
Particle velocity	Magnetic field	E	Wave vectors based on tissue properties (reconstruction plane only)
Intensity	EM energy flow	M/E	Electric field × magnetic field (reconstruction plane only)
Dissipation	Dissipation	E	Dot product of reconstructed electric field and the product of electric field and tissue conductivity

NAH = nearfield acoustic holography, NEH = nearfield electromagnetic holography. EM = electromagnetic M/E = estimated or measured: *Note*: As the reconstruction process is essentially performed *within* a distributed source assignment of physical units to these measurements is not strictly valid.

* One of a number of methods used in NAH.

**Table 2:** Table of formulae.

Characteristic wave number
$\begin{array}{l} k=\sqrt{\hat{\varepsilon }\mu \omega^{2}}\\ \hat{\varepsilon }=\varepsilon \left(1+i\sigma /(\omega \varepsilon )\right) \end{array}$
*σ* is conductivity, *μ* is relative permeability and *ɛ* is relative permeability
$k_{z}=\left\{\begin{array}{lll} \sqrt{k^{2}-k_{x}^{2}-k_{y}^{2}} & \text{for}\;k_{x}^{2}-k_{y}^{2}\leq k^{2} & \left(\text{far-field}\right)\\ -i\sqrt{k_{x}^{2}+k_{y}^{2}-k^{2}} & \text{for}\;k_{x}^{2}+k_{y}^{2}>k^{2} & \left(\text{near-field}\right) \end{array}\right)$
*k*_*x*_ and *k*_*y*_ are spatial wave vectors depending on array layout
Wave vector:
$K=(k_{x},k_{y},k_{z})$
K-space propagator from measurement plane to reconstruction-plane:
$G_{z}=e^{-ik_{z}z}$
*z* is propagation distance
Forward Fourier transform to K-space
$\Psi =F\left(\psi \right)=\int \int_{-\infty }^{\infty }\int \psi e^{ik_{x}x}e^{ik_{y}y}e^{-i\omega t}\text{d}x\text{d}y\text{d}t$
Inverse Fourier transform from K-space
$\psi =F^{-1}\left(\Psi \right)=\frac{1}{{\left(2\pi \right)}^{3}}\int \int_{-\infty }^{\infty }\int \Psi e^{-ik_{x}x}e^{-ik_{y}y}e^{i\omega t}\text{d}k_{x}\text{d}k_{y}\text{d}\omega$
K-space filler:
$\begin{array}{l} K_{filter}=\left\{\begin{array}{c} 1-0.5e^{\left(\left(k_{r}/k_{c}\right)-1\right)/\alpha }\;\text{for}\;k_{r}\leq k_{c}\\ 0.5e^{\left(1-\left(k_{r}/k_{c}\right)\right)/\alpha }\;\text{for}\;k_{r}>k_{c} \end{array}\right)\\ k_{r}=\sqrt{k_{x}^{2}+k_{y}^{2}} \end{array}$
*k*_*c*_ and *α* are filler parameters
Electric field at measurement plane:
E=∇P
*P* is measured potentials
Electric field at reconstruction-plane:
$E_{z}=F^{-1}(F\left(E\right)G_{z}K_{\text{filter}})$
Magnetic field at reconstruction-plane:
$H_{z}=F^{-1}(K\times F\left(E_{z}\right))$
Energy flow poynting vector field at reconstruction-plane:
$S_{z}=E_{z}\times H_{z}$
Energy source density at reconstruction-plane:
${\text{ESD}}_{z}=\nabla \cdot S_{z}$
Energy dissipation at reconstruction-plane: $D_{z}=\sigma E_{z}\cdot E_{z}$
